# Sepsis profile and outcome of preterm neonates admitted to neonatal intensive care unit of Cairo University Hospital

**DOI:** 10.1186/s43054-021-00055-1

**Published:** 2021-03-01

**Authors:** Khaled Salama, Amira Gad, Sarah El Tatawy

**Affiliations:** Neonatal Department, Kasr Al Ainy Hospital, Cairo University, El Saray St Manial, Giza, 11956 Egypt

**Keywords:** Neonatal sepsis, Enterobacteriacea, Antibiotic resistance, NICU, Preterm birth

## Abstract

**Background:**

This study demonstrates the experience of the neonatal intensive care unit (NICU) of a tertiary referral center in Egypt in management of prematures with neonatal sepsis. This retrospective study included preterm neonates admitted to NICU with clinical and/or laboratory diagnosis of sepsis. Blood culture was done followed by antimicrobial susceptibility testing for positive cases. Neonates with sepsis were classified into early onset sepsis (EOS) and late onset sepsis (LOS). Hematological scoring system (HSS) for detection of sepsis was calculated.

**Results:**

The study included 153 cases of neonatal sepsis; 63 (41.2%) EOS and 90 (58.8%) LOS. The majority of the neonates had very low or moderately low birth weight (90.9%). All neonates received first-line antibiotics in the form of ampicillin-sulbactam, and gentamicin. Second-line antibiotics were administered to 133 neonates (86.9%) as vancomycin and imipenem-cilastatin. Mortalities were more common among EOS group (*p* < 0.017). Positive blood cultures were detected in 61 neonates (39.8%) with a total number of 66 cultures. The most commonly encountered organisms were Klebsiella MDR and CoNS (31.8% each). *Klebsiella* MDR was the most predominant organism in EOS (28.9%), while CoNS was the most predominant in LOS (39.2%) The detected organisms were divided into 3 families; *Enterobacteriaceae*, non-fermenters, and Gram-positive family. There 3 families were 100% resistant to ampicillin. The highest sensitivity in *Enterobacteriaceae* and Non-fermenters was for colistin and polymyxin-B. An HSS of 3–8 had a sensitivity and specificity of 62.3% and 57.6%, respectively for diagnosis of culture-proven sepsis.

**Conclusion:**

Neonatal sepsis was encountered in 21.5% of admitted preterm neonates; LOS was more common (58.8%). Mortality was 51.6%. *Klebsiella* MDR and CoNS were the most commonly encountered organisms in both EOS and LOS. The isolated families were 100% resistant to ampicillin. The hematological scoring system (HSS) showed limited sensitivity for detection of sepsis.

## Background

Neonatal sepsis is considered a major cause of morbidity and mortality in neonates, despite marked efforts at moderating its effects [1]. It has been shown that over 4 million babies die in the first 4 weeks of life every year; 3 million of which occur in the early neonatal period [2]. Neonatal infection is very common in preterm infants. Less mature infants who need intensive care and experience invasive procedures are at the highest risk [3].

The diagnosis of infection is usually very difficult in preterm infants. Clinically, the presentation is elusive and nonspecific. The presenting signs include jaundice, difficult breathing and feeding, unstable temperature, and heart rate variability [4]. Diagnosis is further complicated by disease heterogeneity and absent rapid and reliable diagnostic tests [5].

This retrospective study demonstrates the experience of the neonatal intensive care unit (NICU) of Kasr Al-Ainy Hospital, Cairo University, Egypt in cases of premature neonatal sepsis over a period of 1 year from March 2017 to February 2018.

## Methods

This retrospective study was carried out at NICU of a tertiary referral center in Egypt from March 2017 to February 2018. The unit admits inborn patients from the obstetrics department which averages 25,000 deliveries per year. The NICU has a capacity of 45 incubators with a nurse:patient ratio of 1:3. The study included all preterm neonates admitted to the unit during the study period and fulfilling the inclusion criteria. An informed consent was obtained from the legal guardian of every neonate on admission to perform necessary investigations and procedures as needed. All needed official permissions were obtained.

### Inclusion criteria

Inclusion criteria were preterm neonates ≤ 36 weeks’ gestational age with clinical and/or laboratory diagnosis of sepsis. Neonatal sepsis was diagnosed based on the criteria of National Neonatal Forum of India [6]. According to these criteria, two categories were identified, probable (clinical) sepsis and culture positive sepsis.

#### Probable (clinical) sepsis

In an infant having clinical picture suggestive of septicemia, if there is the presence of any one of the following criteria:

- Existence of predisposing factors: maternal fever or foul-smelling liquor or prolonged rupture of membranes (> 24 h) or gastric polymorphs (> 5 per high power field).
- Positive septic screen: presence of two of the four parameters namely, TLC (< 5000/mm), band to total polymorphonuclear cells ratio of > 0.2, absolute neutrophil count < 1800/mm^3^, C-reactive protein (CRP) > 1 mg/dL, and micro ESR > 10 mm-first hour.
- Radiological evidence of pneumonia.

#### Culture positive sepsis

In an infant having clinical picture suggestive of septicemia, pneumonia or meningitis, if there is presence of either of the following:

- Isolation of pathogens from blood or CSF or urine or abscess.
- Pathological evidence of sepsis on autopsy.

### Exclusion criteria

Exclusion criteria were full term neonates and neonates with multiple congenital anomalies. The total number of neonates admitted to the unit during the study period was 1096; 385 full-term and 711 preterm neonates. One-hundred fifty-three neonates fulfilled the eligibility criteria of the study.

### Data collection

Complete history data included gestational age; birth weight; sex; mode of delivery; Apgar score at 1, 5, and 10 min; and length of hospital stay. Complete obstetric history was recorded for detection of risk factors for sepsis as premature rupture of membranes (PROM) > 18 h, chorioamnionitis, maternal urinary tract infection, gestational hypertension, preeclampsia, eclampsia, gestational diabetes, obstructed labor, and multiple gestations.

In-hospital manifestations of sepsis were recorded, such as apnea, feeding difficulties, abdominal symptoms and suspected necrotizing enterocolitis (NEC), poor activity or perfusion, bleeding tendency, temperature instability, sclerema, and convulsions. In addition, antibiotics administered, culture results, medical interventions, presence of intracranial hemorrhage, PDA and pulmonary hypertension, and need for phototherapy or exchange transfusion were recorded.

### Cultures

All neonates had blood culture. Blood samples (3–4 ml) were collected under sterile conditions to be inoculated on BD BACTEC™ Peds Plus™ media. If microorganisms were present, CO_2_ was produced when the organisms metabolize the substrates present in the vial. High amount of CO_2_ increased the fluorescence of the vial sensor, monitored by the BACTEC fluorescent instrument to determine if the vial is positive [7]. Blood was cultured for aerobic organisms only (anaerobic and fungal cultures not done). EOS blood cultures were drawn on day 1 of life (admission) and LOS blood cultures were drawn on day 4 to 7 of admission. Also, 45 neonates had endotracheal tube aspirate culture. Endotracheal cultures were drawn after the first week of life to give a chance for first- and second-line antibiotics. Then, if the neonate was still septic other sources of infection were searched. Neither urine nor CSF cultures were performed for these newborns.

### Antimicrobial susceptibility testing for positive cases

Antimicrobial susceptibility of isolates was determined by the standard Kirby Bauer disk diffusion method using antimicrobial discs (Oxoid limited Basingstoke, Hamsphire and England) stored according to the manufacturer’s instructions. All steps were performed according to the Clinical & Laboratory Standards Institute (CLSI) recommendations, using Muller Hinton agar (Oxoid, Basingstoke, United Kingdom). Disc zone diameters were interpreted according to the CLSI recommendations and categorized according to the breakpoints for disc diffusion testing, as sensitive, resistant or intermediate [8].

Antibiotic sensitivity was assessed for each family of organisms toward the following antibiotics: Cefoxitin, Ceftriaxone, Ceftazidime, Gentamicin, Ampicillin, Imipenem-Cilastatin, Ciprofloxacin, Cefotaxime, Cefuroxime, Tigecycline, Cefoperazone-sulbactam, Trimethoprim-sulfamethoxazole, Cefepime, Linezolid, Colistin, Meropenem, Cefoperazone, Piperacillin-Tazobactam, Vancomycin, Ampicillin-sulbactam, Amikacin, Polymyxin B, Amoxicillin-clavulanic.

Different pathogenic bacteria were identified in blood and endotracheal cultures. Neonates with sepsis were then classified into early-onset sepsis (EOS); signs of sepsis in first 72 h and late-onset sepsis (LOS); signs of sepsis after 72 h. Antibiograms were constructed to detect different antibiotic sensitivity and resistances for common organisms detected in septic newborn babies.

### Hematological scoring system

Hematological scoring system (HSS) of Rodwell et al. (1988) assigns a score of 1 for each of seven findings significantly associated with sepsis. Score 2 is given if there was no mature PMN.

1. Abnormal total leukocyte count; ≤ 5000/mm^3^, ≥ 25,000/mm^3^ at birth, ≥ 31,000/mm^3^ (12–24 h), or ≥ 21,000/mm^3^ (day 2 onwards)
2. Abnormal total PMN count; < 1800/mm^3^ or > 5400/mm^3^, or no mature PMN
3. Elevated immature PMN count (> 600/mm^3^)
4. Elevated immature to total (I:T) PMN ratio (> 0.2)
5. Immature to mature (I:M) PMN ratio (≥ 0.3)
6. Platelet count ≤ 150,000/mm^3^
7. Pronounced degenerative or toxic changes in PMNs

A score of ≤ 2 was interpreted as sepsis unlikely; score 3–4: sepsis is possible and ≥ 5 sepsis is very likely. Minimum score that can be obtained is 0 and maximum score is 8 [9].

## Results

The study included 153 preterm neonates with diagnosis of sepsis; 63 (41.2%) early-onset sepsis (EOS) and 90 (58.8%) late-onset sepsis (LOS). They were 86 males (56.2%) and 67 females (43.8%). Cesarean delivery was more common than vaginal delivery (69.9% versus 30.1%). The majority of the neonates had very low or moderately low birth weight (90.9%). Table 1 demonstrates baseline characteristics of the studied neonates.

**Table 1:** Baseline characteristics of the studied neonates

Variables	All cases *n* = 153	EOS *n* = 63	LOS *n* = 90	*p* value
Gestational age (weeks)	31 ± 2	31 ± 3	31 ± 2	0.035
Male sex	86 (56.2%)	40 (63.5%)	46 (51.1%)	0.129
Birth weight (g)	1490 ±411	1486 ± 455	1494 ± 381	0.91
Incredibly low birth weight (< 750)	1 (0.7%)	1 (1.6%)	0 (0.0%)	0.571
Extremely low birth weight (< 1000)	10 (6.5%)	5 (7.9%)	5 (5.6%)	
Very low birth weight (1000–1499)	74 (48.4%)	31 (49.2%)	43 (47.8%)	
Moderately low birth weight (1500–2499)	65 (42.5%)	24 (38.1%)	41 (45.6%)	
Normal birth weight (2500–3999)	3 (2%)	2 (3.2%)	1 (1.1%)	
SGA	15 (9.8%)	7 (11.1%)	8 (8.9%)	0.666
Multiple gestation	37 (24.2%)	12 (19.0%)	25 (27.8%)	0.215
Cesarean section	46 (30.1%)	20 (31.7%)	26 (28.9%)	0.704
Apgar score				
1 min	3 (0–8)	3 (0–7)	4 (0–8)	0.016
5 min	5 (2–9)	5 (2–9)	6 (2–9)	0.114
10 min	8 (4–10)	7 (5–10)	8 (4–10)	0.061
Duration of mechanical ventilation (days)	7 (0–54)	6 (0-28)	2 (0-54)	0.865
Length of hospital stay	24 ± 22	20 ± 27	26 ± 18	0.132
Deaths	79 (51.6%)	40 (63.5%)	39 (43.3%)	0.014

*EOS* early-onset sepsis, *LOS* late-onset sepsis

Data are presented as mean ± SD, number (%), median (range)

**Fig. 1:**
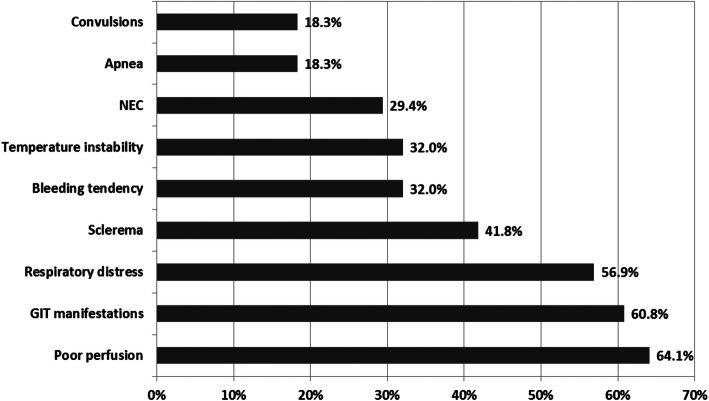
Clinical picture of sepsis in the studied neonates

The most common maternal illnesses were hypertensive disorders of pregnancy (21.6%) and PROM > 18 h (16.3%). Perinatal complications were recorded in 134 cases (87.6%). Respiratory distress was the most common complication followed by apnea (Table 2). Various signs of sepsis were recorded; the most common manifestation was poor activity (77.1%) followed by poor perfusion (64.1%) (Fig. 1). The median duration of mechanical ventilation (MV) was 5 days (range 0–54 days) and that of total parenteral nutrition (TPN) was 11 days (range 0–50 days). The median of hospital stay was 18 days (range 3–198 days).

**Table 2:** Maternal and neonatal illnesses of the studied sample (*n* = 153)

Variables	Value
Maternal diseases	
Preeclampsia/eclampsia, pregnancy induced hypertension	33 (21.6%)
Premature rupture of membranes > 18 hours	25 (16.3%)
Chorioamnionitis	10 (6.5%)
Intrauterine fetal death or abortions	9 (5.9%)
Diabetes mellitus	2 (1.3%)
Obstructed labor	1 (0.7%)
Neonatal morbidities	
Ventilator-associated pneumonia (VAP)	38 (24.8%)
Respiratory distress	120 (78.4%)
Apnea	20 (13.1%)
Hypoglycemia	3 (2.0%)

Data are presented as mean ± SD, number (%), median (range)

All neonates received first-line antibiotics in the form of ampicillin-sulbactam, and gentamicin. Second-line antibiotics were administered to 133 neonates (86.9%) as vancomycin and imipenem-cilastatin. Fifty-four neonates (35.3%) were shifted to a third-line consisted of piperacillin-tazobactam, and ciprofloxacin. Mortality rate was 51.6%. Mortalities were more common among early-onset sepsis group (*p* < 0.017); 39/63 (61.9%) in EOS-onset sepsis vs. 38/90 (42.2%) of LOS.

### Culture results

Positive blood cultures were detected in 61 neonates (39.8%). The total number of positive blood cultures was 66 (all aerobic bacteria) as some cases had more than one positive blood culture. The most commonly-encountered organisms were *Klebsiella* MDR and CoNS (31.8% each) as shown in Table 3. *Klebsiella* MDR was the most predominant organism in EOS (28.9%), while CoNS was the most predominant in LOS (39.2%) as shown in Table 4. Out of the 153 neonates, 38 (24.8%) had positive endotracheal aspirate. The most commonly encountered organism was *Klebsiella* MDR followed by *Acinetobacter* MDR (Table 4).

**Table 3:** Distribution of organisms detected in blood cultures and endotracheal aspirate in the studied neonates

Organism	Number (%)
Blood cultures (*n* = 66)	
*Klebsiella* MDR	21 (31.8%)
CoNS	21 (31.8%)
*Acinetobacter* MDR	8 (12.1%)
MRSA	4 (6.1%)
*Klebsiella* AMPC	3 (4.5%)
*Pseudomonas* MDR	3 (4.5%)
*E*. *coli* MDR	2 (3.1%)
*Klebsiella* ESBL	2 (3.1%)
*Klebsiella oxytoca*	2 (3.1%)
Endotracheal aspirate culture (*n* = 38)	
*Klebsiella* MDR	21 (55.2%)
*Acinetobacter* MDR	9 (23.6%)
*E*. *coli* MDR	2 (5.3%)
Pseudomonas MDR	2 (5.3%)
*Klebsiella oxytoca*	2 (5.3%)
*Klebsiella* spp.	1 (2.6%)
*Klebsiella* AMPC	1 (2.6%)

**Table 4:** Distribution of organisms according to onset of sepsis

Organism	EOS (*n* = 38)	LOS (*n* = 28)
	*n* (%)	*n* (%)
*Klebsiella* MDR	11 (28.9%)	10 (35.7%)
CoNS	10 (26.3%)	11 (39.2%)
*Acinetobacter* MDR	6 (15.8%)	2 (7.1%)
MRSA	2 (5.3%)	2 (7.1%)
*Pseudomonas* MDR	3 (7.9%)	–
*Klebsiella* AMPC	2 (5.2%)	1 (3.6%)
*Klebsiella* ESBL	2 (5.3%)	–
*Klebsiella oxytoca*	2 (5.3%)	–
*E*. *coli* MDR	–	2 (7.1%)

### Antibiograms

Antibiotic sensitivity was calculated for all the studied culture organisms. The detected organisms were divided into 3 families:

- Enterobacteriaceae (all *Klebsiella* species and *E*. *coli* MDR).
- Non-fermenters (*Pseudomonas* MDR, and *Acinetobacter* MDR).
- Gram-positive family (MRSA and CoNS).

These 3 bacterial families were 100% resistant to ampicillin. The highest sensitivity in *Enterobacteriaceae* and non-fermenters was for colistin and polymyxin-B. Table 5 demonstrates antibiotic sensitivity of the 3 main bacterial families.

**Table 5:** Different antibiotic sensitivities of the 3 main bacterial families

	Enterobacteriaceae	Non-fermenters	Gram positive
Cefoxitin	6 (10.5%)	1 (0.4%)	0 (0.0%)
Ceftriaxone	1 (1.5%)	0 (0%)	0 (0.0%)
Ceftazidime	1 (3.0%)	1 (8.3%)	0 (0.0%)
Gentamicin	4 (7.6%)	0 (0.0%)	5 (25%)
Imipenem-Cilastatin	14 (25.0%)	2 (9.5%)	0 (0.0%)
Ciprofloxacin	6 (10.5%)	1 (4.7%)	4 (17.3%)
Cefotaxime	1 (2.0%)	1 (5.5%)	0 (0.0%)
Cefuroxime	2 (3.6%)	0 (0.0%)	–
Cefoperazone-sulbactam	4 (8.0%)	1 (5.2%)	–
Trimethoprim-sulfamethoxazole	5 (9.8%)	2 (0.1%)	4 (20%)
Cefepime	4 (7.5%)	1 (4.3%)	–
Colistin	29 (96.6%)	17 (100.0%)	–
Meropenem	9 (18.7%)	1 (5.8%)	–
Cefoperazone	1 (2.2%)	1 (7.6%)	0 (0.0%)
Piperacillin-Tazobactam	4 (8.3%)	1 (5.8%)	–
Ampicillin-sulbactam	2 (8.6%)	0 (0.0%)	0 (0.0%)
Amikacin	15 (27.7%)	0 (0.0%)	8 (57%)
Polymyxin B	31 (91.1%)	21 (100%)	–
Amoxicillin-clavulanic	3 (5.0%)	1 (4.7%)	0 (0.0%)
Tigecycline	26 (89.6%)	9 (0.6%)	–
Ampicillin	0 (0.0%)	0 (0.0%)	0 (0.0%)
Vancomycin	–	–	24 (100%)
Linezolid			24 (100%)

**Fig. 2:**
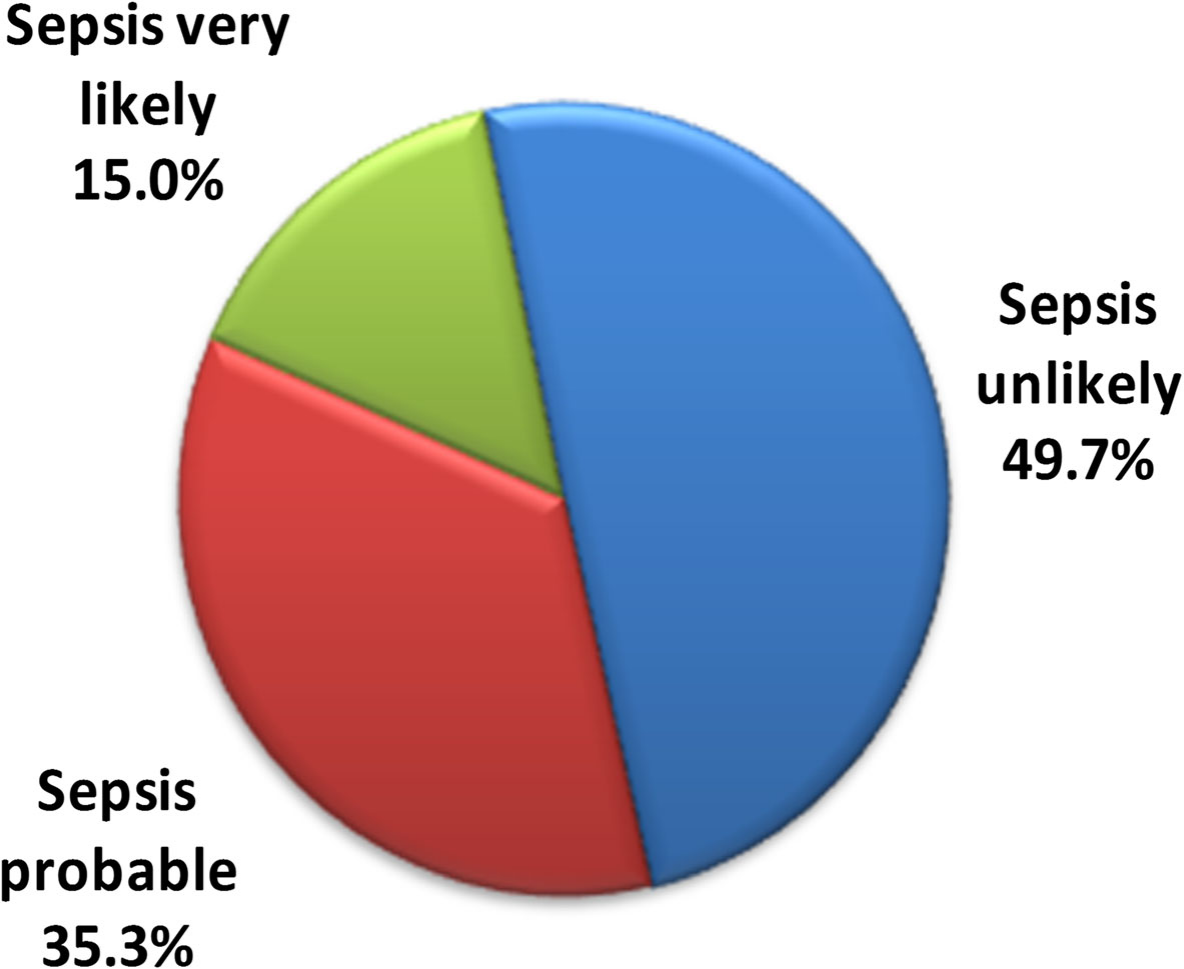
Classification of septic newborn babies using hematological scoring system

Using the hematological scoring system, 76 neonates (49.7%) were classified as sepsis unlikely, 54 (35.3%) as sepsis probable, and 23 (15%) as sepsis very likely (Fig. 2).

Table 6 shows the diagnostic performance of individual items of HSS in detection of early-onset sepsis. The immature to mature PMNs ratio had the highest sensitivity (76.2%), while the immature to total PMNs had the highest specificity (91.1%). On the other hand, individual items did not show good diagnostic performance to detect late-onset sepsis (Table 7). Table 8 shows the diagnostic performance of individual items of HSS in detection of culture-proven sepsis, with immature to total PMNs showing highest specificity (80.4%).

**Table 6:** Diagnostic performance of individual items of HSS for diagnosis of early-onset sepsis

Test	Sensitivity (%)	Specificity (%)	PPV (%)	NPV (%)
Total WBCs	55.6%	70.0%	56.5%	69.2%
Total PMNs	65.1%	53.3%	49.4%	68.6%
Immature PMNs	63.5%	52.2%	48.2%	67.1%
Immature:mature PMNs	76.2%	62.2%	58.5%	78.9%
Immature:total PMNs	44.4%	91.1%	77.8%	70.1%
Platelet count	46.0%	67.8%	50.0%	64.2%

**Table 7:** Diagnostic performance of individual items of HSS for diagnosis of late-onset sepsis

Test	Sensitivity (%)	Specificity (%)	PPV (%)	NPV (%)
Total WBCs	30.0%	44.4%	43.5%	30.8%
Total PMNs	46.7%	34.9%	50.6%	31.4%
Immature PMNs	47.8%	36.5%	51.8%	32.9%
Immature:mature PMNs	37.8%	23.8%	41.5%	21.1%
Immature:total PMNs	8.9%	55.6%	22.2%	29.9%
Platelet count	32.2%	54.0%	50.0%	35.8%

**Table 8:** Diagnostic performance of individual items of HSS for diagnosis of culture-proven sepsis

	Sensitivity (%)	Specificity (%)	PPV (%)	NPV (%)
Total WBCs	41.0%	59.8%	40.3%	60.4%
Total PMNs	62.3%	51.1%	45.8%	67.1%
Immature PMNs	60.7%	50.0%	44.6%	65.7%
Immature:total PMNS	29.5%	80.4%	50%	63.2%
Immature:mature PMNS	57.4%	48.9%	42.7%	63.4%
Platelet count	32.8%	58.7%	34.5%	56.8%

An HSS of 3–8 had a sensitivity and specificity of 62.3% and 57.6% respectively for diagnosis of culture-proven sepsis. The score of 3–8 significantly predicted culture-proven sepsis (*p* = 0.016) with an odds ratio of 2.2 (95% confidence interval: 1.2–4.4).

### Regression models

Concerning relation between risk factors and outcomes, logistic regression model showed that the length of hospital stay (OR: 1.2, 95%CI: 1.1–1.2) and days of mechanical ventilation (OR: 0.87, 95%CI: 0.29–2.6) were the only independent factors predicting death outcome.

## Discussion

This study demonstrated that 21.5% of the preterm neonates admitted to NICU during the study period suffered neonatal sepsis. Among the 153 cases, LOS was more frequent (58.8%). Males constituted 56.2% of premature neonates with proven sepsis. The mortality of the current series was relatively high (51.6%). Mortality was more common among EOS (*p* = 0.017).

The incidence of neonatal sepsis shows a geographical variation. While some reports from developed countries demonstrated that the incidence of neonatal sepsis varies from 1 to 5 cases per 1000 live births, some other population-based studies from developing countries have reported clinical sepsis rates ranging from 49 to 170 per 1000 live births [10]. In Africa, the reported incidence varies from 6.5 to 23 per 1000 live births [11]. The wide variability can be contributed to the differences in socioeconomic levels, perinatal and neonatal care facilities, infection control protocols, and antibiotic use. In the current study, neonatal sepsis affected 21.4% of this specific group of preterm neonates admitted to the NICU over 1-year period. Similar studies in Egypt reported higher rate around 33% among neonates admitted to NICU [12, 13]. Another study of neonates admitted to NICU reported sepsis rates of 20.5% [14]. Studies from Brazil and Indonesia reported higher rates of infections among NICU admissions up to 46–51% [15, 16].

Among the 153 cases, LOS was more frequent (58.8%). This is in agreement with previous studies from different countries [17–19]. Previous Egyptian studies reported comparable proportions of 60.8% [12] and 64.6% [13] of LOS in neonates admitted to NICU. On the contrary, EOS was more common in a study conducted in an NICU in Kanpur, India [20]. This was confirmed in more recent studies from another developing country (Nepal). Early-onset sepsis accounted for 84% in one study [21] and 78.3%in another one [14]. Males constituted 56.2% premature neonates with proven sepsis. This goes in concordance with previous studies [22–24].

The gold standard of diagnosis of neonatal sepsis is blood culture for isolation of the causative pathogen. Nevertheless, failure of growing the pathogenic microorganism in culture is common due to many reasons [25]. Consequently, clinical and laboratory diagnostic methods are recommended to be added to blood culture for the diagnosis of neonatal sepsis [26]. In the current study, the patients were diagnosed as probable or clinical sepsis based on the criteria of National Neonatal Forum of India [6]. Among the 153 neonates with clinical sepsis, blood cultures were positive in 61 neonates (39.8%). This rate is similar to rates reported in Egypt (40.7%) [24] and many developing countries [27–29]. In studies involving neonates admitted to the NICU, culture-positive sepsis was proved in variable proportions between 20.5% and 42.8% [12–14, 20].

The most commonly encountered organisms in the current study were *Klebsiella* MDR (31.8%) and CONS (31.8%). Gram-negative bacilli constituted the majority of isolated organisms (62%). This distribution of organisms is the same in cases with EOS and LOS. Pokhrel et al. reported that 77% of bacterial isolates in their series of NICU admitted neonates were Gram-negative [14]. An Egyptian study reported Gram-negative bacteria in 68% of positive cultures [13]. Similar to the current study, *Klebsiella* species and CoNS were the most common [12, 14]. Gram-negative organisms constituted 61% of isolates with *Klebsiella* being the most common organism in another study [21]. *Klebsiella pneumoniae* ranked second after *Staphylococcus aureus* in a study of neonates admitted to NICU in India [20].

A previous Egyptian study reported that Gram-positive cocci, specifically CoNS, were more commonly isolated compared to Gram-negative organism [24]. Similar findings were obtained in other studies [30–32]. These results were consistent with a review of 11471 bloodstream isolates from developing countries, where *Klebsiella pneumoniae*, other Gram-negative rods (*E*. *coli*, *Pseudomonas* spp., *Acinetobacter* spp.), and *Staphylococcus aureus* were the major isolated pathogens. This review included hospital-born neonates [33]. Predominance of Gram-negative organisms may be due to the inappropriate and unselective use of antibiotics in addition to deficiency of hygienic practice during delivery and newborn handling [13]. The most commonly isolated agents in studies conducted in Western countries were group B streptococci (GBS) followed by Gram-negative bacilli and staphylococci [30].

In the current study, the 3 isolated bacterial families (*Enterobacteriaceae*, non-fermenters, and Gram-positive) were 100% resistant to ampicillin. Mohsen et al. reported similar antibiotic susceptibility pattern of the isolated Gram-negative bacilli that showed highest resistance to ampicillins, cephalosporins, and piperacillin-tazobactam [12]. Another Egyptian study showed high resistance of both Gram-positive and Gram-negative bacteria against the common antibiotics in use including ampicillin, amoxicillin, cefotaxime, ceftriaxone, and gentamicin [13]. The high resistance to these common antibiotics was reported in other studies from Egypt and other developing countries [14, 20, 21, 34].

In the current study, *Klebsiella* MDR and *Acinetobacter* MDR constituted more than 40% of the isolated organisms. Bacteria causing neonatal sepsis have developed increased resistance to commonly used antibiotics which expand the difficulty of the problem to include management as well as diagnosis due to emergence of MDR organisms as important pathogens causing sepsis in the NICU [35]. A retrospective review from Jordan reported that MDR organisms constituted 69% of sepsis episodes in NICU [36]. Other developing countries reported the emergence of MDR organism [37–39]. Optimizing antimicrobial use seems to be the key to reduce antimicrobial resistance. In Panama, resistance rates started to be lower after initiation of a program to stop empiric antibiotics after 3 days if neonates were improving with normal laboratory markers of infection and negative cultures [40].

The most common maternal illnesses were hypertensive disorders of pregnancy (21.6%) and PROM > 18 h (16.3%). Perinatal complications were recorded in 134 cases (87.6%). Respiratory distress was the most common complication followed by apnea (Table 2).

The mortality of the current series was relatively high (51.6%). Mortality was more common among EOS (*p* < 0.017). The Egyptian study conducted in Mansoura University reported 51% mortality from EOS and 42.9% for LOS coinciding with the results of the present study; this may be explained by the fact that both hospitals are busy tertiary referral centers admitting large number of preterm babies. On the contrary, mortality rate was only 12% in a study from a Nigerian private tertiary hospital that included more term than preterm neonates [41].

In the current study, we tested the diagnostic accuracy of hematological scoring system (HSS) for early detection of neonatal sepsis. The difficult and delayed diagnosis and the high fatality of neonatal sepsis adds to the importance of finding a reliable laboratory tool for early diagnosis and management of this fatal condition. Unfortunately, HSS was of moderate sensitivity for detection of sepsis (62.3%). The immature to mature PMNs ratio had the highest sensitivity (76.2%), while the immature to total PMNs had the highest specificity (91.1%). Narasimha and Kumar reported similar findings in a small sample of neonates [42]. These results are contradictory to that of Makkar et al. who reported sensitivity and specificity of HSS of 92.3% and 86.4%, respectively for detection of neonatal sepsis in premature infants [9].

## Conclusion

Neonatal sepsis was encountered in 21.5% of all preterm neonatal admissions to the NICU comprising higher proportion of LOS (58.8%) with high mortality of 51.6%; more common among EOS (*p* = 0.017). *Klebsiella* MDR and CoNS were the most commonly encountered organisms in both EOS and LOS, respectively. The 3 isolated bacterial families (Enterobacteriaceae, non-fermenters, and Gram-positive) were 100% resistant to ampicillin. The hematological scoring system (HSS) showed limited sensitivity for detection of sepsis.

## Data Availability

The datasets analyzed during the current study are available from the corresponding author on reasonable request.

## Abbreviations

CLSI: Clinical & Laboratory Standards Institute
CoNS: Coagulase-negative staphylococci
EOS: Early-onset sepsis
GBS: Group B streptococci
HSS: Hematological scoring system
LOS: Late-onset sepsis
MDR: Multi-drug resistant
MRSA: Methicillin-resistant *Staphylococcus aureus*
MV: Mechanical ventilation
NEC: Necrotizing enterocolitis
NICU: Neonatal intensive care unit
PMN: Polymorphonuclear neutrophils
PROM: Premature rupture of membranes
TPN: Total parenteral nutrition
